# Esmolol infusion in patients with septic shock and tachycardia: a prospective, single-arm, feasibility study

**DOI:** 10.1186/s40814-018-0321-5

**Published:** 2018-08-03

**Authors:** Samuel M. Brown, Sarah J. Beesley, Michael J. Lanspa, Colin K. Grissom, Emily L. Wilson, Samir M. Parikh, Todd Sarge, Daniel Talmor, Valerie Banner-Goodspeed, Victor Novack, B. Taylor Thompson, Sajid Shahul, The Esmolol to Control Adrenergic Storm in Septic Shock-ROLL-IN (ECASSS-R) study, Naresh Kumar, Brent Armbruster, Valerie Aston, Anne Haroldsen

**Affiliations:** 1 Pulmonary and Critical Care Medicine, Intermountain Medical Center, Murray, UT USA; 2 Pulmonary and Critical Care Medicine, University of Utah, Salt Lake City, UT USA; 3 Nephrology and Vascular Biology, Beth Israel Deaconess Medical Center, Boston, MA USA; 4 Anesthesia and Critical Care Medicine, Beth Israel Deaconess Medical Center, Boston, MA USA; 5 Pulmonary and Critical Care Medicine, Massachusetts General Hospital, Boston, MA USA; 6 Department of Anesthesia, University of Chicago, Chicago, IL USA; 7 Shock Trauma Intensive Care Unit, Intermountain Medical Center, 5121 South Cottonwood Street, Murray, UT 84107 USA

**Keywords:** Sepsis, Beta blockade, Adrenergic antagonism, Clinical trial, Heart rate variability, Organ-failure-free days, Multiple organ dysfunction

## Abstract

**Background:**

High adrenergic tone appears to be associated with mortality in septic shock, while adrenergic antagonism may improve survival. In preparation for a randomized trial, we conducted a prospective, single-arm pilot study of esmolol infusion for patients with septic shock and tachycardia that persists after adequate volume expansion.

**Methods:**

From April 2016 to March 2017, we enrolled patients admitted to an intensive care unit with sepsis who were receiving vasopressor infusion and were tachycardic despite adequate volume expansion. All patients received a continuous intravenous infusion of esmolol, targeted to heart rate 80–90/min, while receiving vasopressors. The feasibility outcomes were proportion of eligible patients consented, compliance with pre-infusion safety check, and compliance with the titration protocol. The primary clinical outcome was organ-failure-free days (OFFD) at 28 days.

**Results:**

We enrolled 7 of 10 eligible patients. Mean age was 46 (± 19) years, and mean admission APACHE II was 28 (± 8). Median norepinephrine infusion rate at the initiation of esmolol infusion was 0.20 (0.14–0.23) μg/kg/min. Compliance with the safety check was 100%; compliance with components of the titration protocol was 98–100%. OFFD were 26 (24.5–26); all patients survived to day 90. Median peak esmolol infusion was 50 (25–50) μg/kg/min. Median peak norepinephrine infusion rate during esmolol infusion was 0.46 (0.13–0.50) μg/kg/min. Four patients achieved target heart rate. Protocol-defined stop events, suggesting possible intolerance to a given infusion rate, occurred in three patients, all of whom were receiving at least 50 μg/kg/min of esmolol.

**Conclusions:**

In a pilot, single-arm study, we report the first published experience with esmolol infusion in tachycardic patients with septic shock in the United States. These findings support a phase 2 trial of esmolol infusion for septic shock. Lower infusion rates of esmolol infusion may be better tolerated and more feasible than higher infusion rates for such a trial.

**Trial registration:**

This study was retrospectively registered at ClinicalTrials.gov (NCT02841241) on 19 July 2016.

**Electronic supplementary material:**

The online version of this article (10.1186/s40814-018-0321-5) contains supplementary material, which is available to authorized users.

## Background

Septic shock, a common syndrome [1] in which infection leads to potentially fatal disruption of homeostasis, accounts for 10% of all ICU admissions and 30% of all ICU mortality [2]. Despite recent improvements in mortality for sepsis, [3] hospital mortality for patients with septic shock remains 22–50% [4, 5]. In septic shock, extreme biological stress is associated with rapidly evolving hemodynamic, physiologic, and metabolic dysfunction, as the host attempts to destroy the invading microorganism, repair damaged tissues, and reestablish homeostasis [6, 7]. The resulting disarray is severe, and cardiovascular dysfunction resulting from septic shock is often life-threatening [8].

While immune activation and autonomic nervous system stimulation are crucial for the host to combat infection, these adaptive responses can become exaggerated and pathogenic. Sympathetic overstimulation can drive a positive feedback loop of cardiovascular and other organ dysfunction, called the multiple organ dysfunction syndrome (MODS), which is the signature of septic shock [8, 9]. The hemodynamic changes of septic shock result from interactions among cardiovascular homeostasis, the host immune response, and therapeutic interventions [6]. These aspects of sepsis are likely interdependent [10]. Nevertheless, administration of exogenous catecholamines to maintain arterial blood pressure has been a cornerstone of management of septic shock for decades. Given this ongoing reliance on catecholamine infusions, recent possible improvements (admitting that some dispute these improvements [1]) in sepsis mortality may be difficult to interpret [3, 11].

Catecholamines have a direct cardiotoxic effect through oxidative damage on the myocardial membrane, including acute myocardial contraction band necrosis and cellular apoptosis [12–14]. A trial using dobutamine to increase cardiac output in sepsis led to increased mortality [15]. In an observational cohort, catecholamine use was associated with increased mortality in septic shock, even after adjusting for disease severity and propensity to receive catecholamines [16]. Similarly, maintenance of normal heart rate—a marker of low sympathetic tone, high parasympathetic tone, or both—is associated with better survival in septic shock [17].

Investigators and clinicians have become interested in β-adrenergic blockade in septic shock [18–21]. A pilot, randomized, open-label trial of esmolol infusion in tachycardic patients with septic shock demonstrated safety and was associated with decreased mortality, [22, 23] although the control-group in-hospital mortality was 91%, much higher than predicted by current severity scores [24]. Experience from small trials in Europe and Asia has largely focused on esmolol protocols targeted to heart rate thresholds. Study designs have assumed that tachycardia per se is injurious in septic shock due to impaired central hemodynamics, even though in heart failure patients β-adrenergic receptor blockade may also improve cardiac performance through neurohormonal mechanisms regardless of heart rate [25–27].

Documented safety and the possibility of efficacy suggest the importance of further investigation of β-adrenergic blockade in septic shock. We therefore undertook a pilot study to assess feasibility of protocols for intravenous esmolol infusion in patients with septic shock with tachycardia that persists after adequate volume expansion.

## Methods

### Patient selection

In this prospective, single-arm, pilot study, we administered esmolol (BREVIBLOC™) infusion to patients who met study eligibility criteria (see Additional file 1: Table S1) and provided written informed consent. Patients were enrolled in the Shock Trauma Intensive Care Unit (a 24-bed multidisciplinary ICU) of Intermountain Medical Center, in Murray, Utah, USA. We enrolled from April 2016 to March 2017. Briefly, patients had septic shock and persistent tachycardia (heart rate > 90/min) after adequate volume expansion. Patients with cardiogenic shock or other severe cardiac disease were excluded (Additional file 1: Table S1). The adequacy of volume expansion was assessed before enrollment (see Additional file 1: Table S2 for the protocol employed) on the basis of clinically obtained assessments; the adequacy of volume expansion was then confirmed using quantitative metrics of fluid responsiveness before esmolol infusion. We defined septic shock according to consensus definitions current at the time of study launch, as two or more markers of the Systemic Inflammatory Response Syndrome (SIRS) plus hypotension requiring vasopressor infusion [28]. Because the study was initiated before the publication of SEPSIS-3 guidelines, [29] we did not stipulate lactate testing or require a specific lactate level. Patients were identified through investigator and/or coordinator review of ICU admission logs with the intent to screen every patient admitted with septic shock to the study ICU.

Two populations are likely at increased risk of intolerance to esmolol infusion: patients who are not adequately volume expanded and patients who have cardiogenic shock. Therefore, all patients underwent a safety check (see Additional file 1: Figure S1 for protocol) after providing consent but before esmolol infusion to assure that volume expansion was adequate and that cardiogenic shock was not present.

After the safety check, esmolol infusion was titrated according to protocol (see Additional file 1: Appendix 2 for details), with a target heart rate of 80–90/min. Esmolol was up-titrated by 5–20 μg/kg/min per step to a maximum rate of 100 μg/kg/min as frequently as every 20 min but not during a volume status assessment or vasopressor up-titration. Esmolol infusion was continued until 3 h after vasopressors had been stopped or until heart rate fell below 80/min. As part of the titration protocol, esmolol infusion could trigger either stop events (which defined possible intolerance of esmolol infusion at a given infusion rate) or volume status assessment events (which required reevaluation of the adequacy of volume expansion). We recommended but did not require a specific vasopressor titration strategy. The study ICU provides bundle-compliant care, [30] including early antibiotics, source control, and prompt attention to adequacy of circulation, as reflected in the current Surviving Sepsis recommendations [31].

### Outcomes

The pre-specified feasibility outcomes were proportion of eligible patients consented, compliance with pre-infusion safety check, and compliance with titration protocols. We also measured the time required for esmolol initiation. Protocol compliance outcomes were defined as in Additional file 1: Table S3. The pre-specified primary clinical outcome was organ-failure-free days (OFFD). As of day 28 after enrollment, the OFFD represents the number of calendar days on which no organ dysfunction was present, as defined by absence of vasopressor therapy, renal replacement therapy, and mechanical ventilation, following the philosophy of the UK Critical Care minimum dataset standards [32] as implemented in several recent trials [33–35]. We employed the “last off” method, by which only organ-failure-free days after the last liberation from all life support therapies are counted as organ-failure free. Patients who died on or before day 28 were assigned − 1 organ-failure-free days in order to avoid equating death with prolonged organ failure. Key secondary clinical outcomes included 28-day all-cause mortality and 90-day all-cause mortality. The conclusion of the study for each patient was at 90 days after enrollment.

### Clinical data

We measured Acute Physiology, Age, and Chronic Health Evaluation score, version 2 (APACHE II) [36] and sequential organ failure assessment (SOFA) [37] scores, receipt of mechanical ventilation, vasopressor infusion rates, and baseline lactate levels, where clinically available. Vasopressor infusion rates were expressed as norepinephrine-equivalent infusion rates, according to our published method [38]. We used a validated [39–41] bioreactance stroke-volume monitoring device to continuously monitor cardiac index. Echocardiograms were performed at the time of study enrollment and 24 h later by trained and credentialed cardiac sonographers according to study protocol. Left ventricular global longitudinal strain was measured from the apical 2, 3, and 4 chamber views and calculated using the validated Tomtec™ software [42] (Further details are reported in Additional file 1: Appendix 1.).

We also measured heart rate variability, as discussed in Additional file 1: Appendix 1. We also measured arterial elastance, estimated by the ratio of mean arterial pressure to stroke volume at the time of echocardiography [43, 44].

To understand protocol intensity and feasibility, we measured the amount of investigator and research coordinator time required for the initial titration phase and discussed experience with the protocol with clinical nursing and physician staff.

### Statistical methods

Among esmolol-treated patients, we compared esmolol-tolerant to esmolol-intolerant patients as a difference of medians, using Wilcoxon’s rank-sum test to compare medians and Chi-square or Fisher’s exact test to compare frequencies. Significance level (two tailed) was 0.05. All analyses were conducted using the R Statistical Package [45]. No formal sample size calculations were performed for this roll-in pilot study, which anticipated enrollment of 10 patients with the expectation that this would provide adequate experience with study protocols.

We also estimated the average effect of the initial esmolol infusion rate on (1) mean arterial pressure, (2) norepinephrine infusion rates, (3) heart rate, and (4) cardiac index, standardizing to the effect of 10 μg/kg/min.

### Ethics, consent, and permissions

This study was registered with clinicaltrials.gov (NCT02841241) and was approved by the Intermountain Medical Center Institutional Review Board (1050147). Written informed consent was obtained from all patients or their legally authorized representatives. When possible (i.e., after the patient recovered capacity), written informed re-consent was obtained directly from the patient. The study was overseen by a safety monitoring committee, which made a formal review of patients at *n* = 3, *n* = 6, and study conclusion.

## Results

**Fig. 1:**
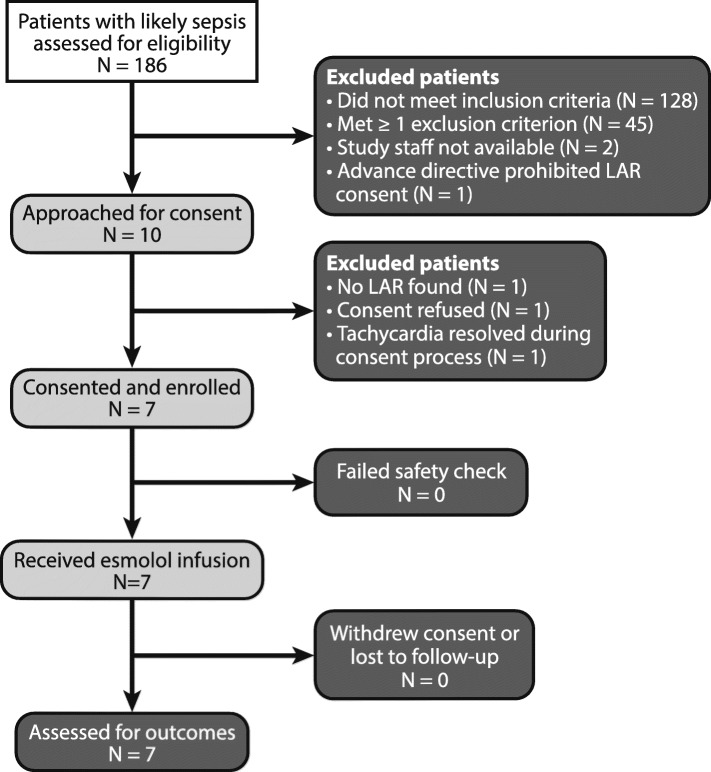
Flow of patients screened and enrolled in the present study

Of 186 patients suspected to have sepsis, 87 patients had septic shock (Fig. 1). We excluded 78 patients with sepsis mimics (e.g., drug overdose, pancreatitis, or metabolic disarray), 21 patients without shock, 20 patients without tachycardia, and 9 for either lack of an arterial catheter or being out of the enrollment window. Among the 10 eligible patients approached, 7 consented and enrolled; all 7 patients completed a 90-day follow-up.

Patient attributes at study enrollment are displayed in Table 1. Mean age was 46 (± 19) years, and mean admission APACHE II was 28 (± 8). Median norepinephrine-equivalent infusion rate at the initiation of esmolol infusion was 0.20 (0.14–0.23) μg/kg/min (two patients were also receiving vasopressin infusion at 0.03 units/min). Two of seven (29%) patients were mechanically ventilated. All patients had sepsis-induced hypotension; six of seven (86%) patients met SEPSIS-3 criteria for septic shock on the basis of serum lactate > 2 mmol/L. No patients received anti-arrhythmic medications before or during esmolol infusion.

**Table 1:** Patient attributes at beginning of esmolol infusion

Variable	Esmolol-treated patients (*n* = 7)
Age (years)	46 (± 19)
Female sex	5 (71%)
Cause of sepsis *n* (%)	
Pneumonia	2 (29%)
Skin/soft tissue	3 (43%)
Urinary source	1 (14%)
Abdominal	1 (14%)
Duration of vasopressor therapy (hours)^a^	15.1 (± 9)
Norepinephrine infusion rate (μg/kg/min)	0.20 (± 0.09)
Receiving vasopressin *n* (%)	2 (29%)
Heart rate (/min)	109 (± 15)
Mean arterial pressure (mmHg)	71 (± 7)
Lactate (mmol/L)^b^	4.8 (± 3.3)
Intravenous crystalloid prior to enrollment (L)	3.5 (3.4–9.0)
Admission APACHE II score (points)	28 (± 8)
Admission SOFA score (points)	11 (± 2)

Values are reported as central tendency and variation, expressed as mean (± standard deviation) or median (inter-quartile range), as appropriate

^a^At time of initiating esmolol infusion

^b^Peak lactate on day of enrollment

Protocol compliance was 100% for the safety check, 100% for stop events, and 98% for the titration protocol, which met or exceeded all protocol-specified thresholds. A physician investigator and clinical research coordinator were present during the titration phase for all patients. Nurses and clinical research coordinators subjectively reported a high burden associated with evaluation of frequent esmolol up-titrations and clinical time required to perform volume expansion events and vasopressor titrations.

All esmolol-treated patients survived to hospital discharge and 90 days. Median (IQR) OFFD among esmolol-treated patients was 26 (24.5–26) days. Clinical outcomes among esmolol-treated patients are displayed in Table 2. By way of contextualization, among 278 patients with septic shock and tachycardia enrolled in recent observational sepsis studies at the same center, [46–48] the hospital mortality was 26%, representing an APACHE II standardized mortality ratio of 0.37 (95% CI 0.29–0.46).

**Table 2:** Clinical outcomes

Variable	Esmolol-treated patients (*n* = 7)
OFFD among all patients (units)	26 (24.5–26)
OFFD among 28-day survivors (days)	26 (24.5–26)
ICU LOS among survivors (days)	3.3 (3.1–5.4)
Hospital LOS among survivors (days)	8.2 (7.1–17.3)
Mortality	
In-hospital mortality, *n* (%)	0 (0%)
28-day all-cause mortality, *n* (%)	0 (0%)
90-day all-cause mortality, *n* (%)	0 (0%)

*OFFD* organ-failure-free days, *ICU* intensive care unit, *LOS* length of stay

All patients passed the safety check (see Additional file 1: Table S4). In one patient, 3.75 L and in another 1 L of crystalloid (administered as fluid boluses of 250–500 mL infused over 5 min) were required to achieve adequate volume expansion as part of the safety check. In the other five patients, the safety check confirmed the pre-enrollment adequacy of volume expansion.

Esmolol was initiated 15 (± 9) hours after initiation of vasopressor infusion. Two patients were enrolled > 24 h after initiation of vasopressor infusion; four patients were enrolled > 12 h after initiation of vasopressor infusion. The titration phase lasted 4.8 (2.8–6.9) hours. Four (57%) patients achieved target heart rate. Mean overall duration of esmolol infusion was 13 (± 10) hours, while mean duration of vasopressor infusions was 49 (± 29) hours.

While undergoing esmolol infusion, cardiac index, heart rate, and vasopressor infusion rate (in norepinephrine-equivalent doses) changed over time as depicted visually in Additional file 1: Figure S2 and S3. Mean stroke volume index at initiation of esmolol infusion was 34 (± 9) mL/m^2^, with mean cardiac index of 3.7 (± 1) L/min/m^2^. With regard to the initial infusion rates (which ranged from 5 to 20 μg/kg/min), a 10 μg/kg/min initial esmolol infusion rate was associated with a 4 mmHg decrease in MAP and a 6 min^−1^ decrease in heart rate (see Additional file 1: Table S5 for further details). No patients developed bradycardia during esmolol infusion; one patient experienced atrial fibrillation after completion of esmolol infusion.

Total intravenous crystalloid administered for volume expansion events while undergoing esmolol infusion was 0.5 (IQR 0–2.5) L, which represented a mean of 2.2 (range 0–7) graded volume expansion challenge interventions. No patients experienced symptomatic fluid overload (e.g., pulmonary edema related to left atrial hypertension).

Echocardiographic results are displayed in Additional file 1: Table S6. On the initial echocardiogram, left ventricle (LV) global longitudinal strain (GLS) was impaired, with median of − 11.5 (− 19.6 to − 9.4)%, while left ventricular ejection fraction (LVEF) was largely normal, with median of 59 (48–53)%. The subsequent day 1 LV GLS was numerically improved (median − 15.8%), although small numbers limited the comparison (see Additional file 1: Figure S4 for details); day 1 LVEF was numerically equivalent. Troponin levels were median 0.21 (0.10–0.87) ng/mL at time of enrollment and 0.14 (0.12–0.47) ng/mL 24 h later. Arterial elastance was 1.2 (1.0–1.6) mmHg/mL at the time of the initial echocardiogram and was numerically higher at the time of the day 1 echocardiogram 1.6 (1.3–1.8; *p* = 0.4) mmHg/mL. Heart rate variability parameters (see Additional file 1: Table S7 for details) did not appreciably change between esmolol initiation and follow-up (at time of esmolol discontinuation or 24 h, whichever was shorter).

Esmolol infusion was discontinued for prespecified stop events indicating possible intolerance in three patients. In one patient (peak esmolol infusion 100 μg/kg/min; 10 h of esmolol infusion), an ScvO_2_ obtained as part of usual clinical care was 50%, with decreasing norepinephrine infusion rate and preserved urine output. Within 20 min of esmolol discontinuation, MAP rose from 75 to 84 mmHg and heart rate rose from 97 to 103 min^−1^. Repeat ScvO_2_ 60 min after esmolol discontinuation was 65%. In one patient (peak esmolol infusion 50 μg/kg/min; 2 h of esmolol infusion), in the transition from 40 μg/kg/min to 50 μg/kg/min of esmolol, the cardiac index decreased briefly (< 15 min) from 2.2 to 1.9 L/min/m^2^ in the setting of higher norepinephrine infusion rate (from 0.33 to 0.42 μg/kg/min). Volume expansion assessment was indicated, but the clinical nurse reported that the assessment would take too long to complete; a stop event was therefore activated by the physician investigator. Esmolol was discontinued, and within 20 min, the cardiac index rose to 2.9 L/min (it had been 2.7 L/min before initiation of esmolol infusion), mean arterial pressure rose from 60 to 69 mmHg, and heart rate rose from 96 to 106 min^−1^. In a third patient (peak esmolol infusion 50 μg/kg/min; 8 h of esmolol infusion), LVEF had worsened slightly (from 46% to approximately 35%) on the basis of a bedside echocardiogram, and the clinical attending physician requested initiation of an inotrope infusion, which triggered a stop event. The patient had no symptoms related to the apparent decrease in LVEF. Within 20 min of esmolol discontinuation, MAP rose from 66 to 71 mmHg and heart rate rose from 93 to 103 min^−1^; repeat bedside echocardiogram suggested that LVEF had returned to approximately 45%.

In each case, esmolol infusion was not resumed for logistical reasons (i.e., need for nurse to attend to other tasks), with patients no longer meeting indications for esmolol by the next day due to clinical improvement. In comparing patients with and without esmolol intolerance, potentially distinguishing factors included peak esmolol infusion rate, stroke volume before esmolol initiation, and arterial elastance before esmolol initiation (see Additional file 1: Table S8 for details).

## Discussion

We performed a pilot feasibility study of esmolol infusion for patients in septic shock with persistent tachycardia after adequate volume expansion. While several small trials have administered β-blockers to just over 250 patients with septic shock (details summarized in Table 3), [22, 23, 49–54] we report here, to our knowledge, the first published experience with esmolol infusion in tachycardic patients with septic shock in the US. Compliance with the protocol was high, and the protocol identified some patients with possible intolerance to higher infusion rates of esmolol. Our study is neither intended nor powered to support inferences about the efficacy of esmolol for clinical outcomes.

**Table 3:** Published experience with esmolol infusion in septic shock

Hospital/country	Patients receiving esmolol (*n*)	Wait 24 h^b^	Peak infusion rates (μg/kg/min)^a^	Esmolol mortality (%)	Use of non-adrenergic inotropes	RCT^d^
Intermountain/USA	7	No	50 (25–50)	0	No	No
La Sapienza/Italy [23]	77	Yes	22 (11–66)	49	Yes	Yes
La Sapienza/Italy [22]	26	Yes	55 (22–233)	NA^c^	Likely^c^	No
La Sapienza/Italy [44]	45	Yes	NA^c^	51	Likely^c^	No
Prague/Czech Rep [49]	10	No	61 ± 20	10	No	No
Peking Union/China [54]	63	No	25 ± 20	8	No	No
Jiangxi/China [53]	30	No	NA^c^	40	Yes	Yes

^a^We assumed 75 kg body mass where unindexed rates provided

^b^Patients were enrolled only > 24 h after onset of septic shock

^c^Authors did not reply to email request for this data

^d^Utilized a randomized controlled design

Clinical and research staff reported that the esmolol titration protocol was time-intensive—almost 5 h required for the titration phase—suggesting potential infeasibility for multi-center application. These observations from clinical staff dovetailed with observations about possible intolerance of esmolol infusion at higher infusion rates. Three of four patients undergoing esmolol infusion at a rate of at least 50 μg/kg/min met criteria for a stop event indicating possible intolerance. None of the three patients receiving infusion rates < 50 μg/kg/min met criteria for a stop event or showed other evidence of intolerance to esmolol infusion. We thus chose to stop enrollment at seven patients in the present pilot study.

We acknowledge the risk of spurious inferences from small numbers, coupled with the necessity of safe and ethical performance of research. Given our experience reported here, we suggest that lower maximum infusion rates may be more feasible and better tolerated than higher infusion rates of esmolol. Lower maximum infusion rates would also be compatible with observations from treatment of cardiac failure suggesting the relevance of neurohormonal mechanism independent of direct hemodynamic effects of beta blockade. [25–27].

Our findings should be placed in the context of prior experience with esmolol in patients with septic shock and tachycardia (see Table 3). The largest prior trial, performed by Morelli et al., randomized 77 patients to esmolol infusion [23]. Patients were enrolled after 24 h of vasopressor therapy for septic shock, when heart rate > 95/min. Acknowledging that length of stay at their study hospital may exceed typical United States (US) hospital length of stay, in-hospital mortality among control (91%) and esmolol-treated patients (68%) was higher than normally observed in the US. Even if assuming that 28-day mortality at their study hospital was analogous to US hospital mortality, the mortality among esmolol-treated patients (49%) was comparable to a large cohort of US patients who did not receive esmolol but would have met their study eligibility criteria, as we have previously demonstrated [24]. Also in distinction from the results of Morelli et al., we observed some increases in norepinephrine infusion rates and decreases in stroke volume with esmolol infusion.

Our research design differed from that of Morelli et al. in important ways. First, we did not administer levosimendan, which was used as rescue therapy in 49% of esmolol-treated patients (40% of controls) by Morelli et al. Levosimendan, a calcium sensitizer that augments left ventricular inotropy and tends to induce tachycardia, does not improve organ function or outcomes on its own in sepsis [55]. Levosimendan likely masked intolerance by offsetting decreases in cardiac contractility related to esmolol (a small Chinese study [53] employed milrinone infusion with likely similar effects). Second, we enrolled patients as soon as volume expansion was judged adequate by objective criteria, with an average lapsed time between vasopressor initiation and esmolol initiation of 15 h; whereas, Morelli et al. enrolled patients after 24 h of vasopressor infusion. This is not, in our view, an important source of difference given our protocol-based, quantitative monitoring and confirmation of the adequacy of volume expansion, based on current best-practice measures of fluid responsiveness. In fact, despite the 24-h optimization, patients treated with esmolol in the Morelli et al. study still received 5.0 (4.3–5.4) L volume expansion over the subsequent 24 h, suggesting that those patients may not have been adequately volume expanded at the time of enrollment or, on the other hand, the patients may have been hypervolemic. Our patients, by contrast, received 0.5 (0–2.5) L of fluid during esmolol infusion, guided by evidence-based evaluation of fluid responsiveness. Third, we employed a formal protocol for frequent reassessment of the adequacy of volume expansion using contemporary dynamic parameters of fluid responsiveness, while Morelli et al. employed static measures (pulmonary artery occlusion pressure and central venous pressure) of fluid responsiveness, making comparison difficult. Fourth, we enrolled patients at a threshold of heart rate > 90/min. Notably, though, we only enrolled one patient who would have been ineligible by the Morelli et al. heart rate criteria (> 95/min). Fifth, the esmolol-treated mortality in our cohort (and, contextually, the mortality among similar patients at our study hospital) was much lower than that observed in the Morelli et al. cohort; this was not apparently due primarily to a lesser severity of illness but to higher survival for a given severity of illness among all patients. Sixth, we do not use pulmonary artery catheters and did not observe substantial rates of drug-resistant infections, which also distinguishes our patients from those reported in the Morelli trial. While Morelli et al. did not report weight-adjusted infusion rates, assuming an average weight of 75 kg, the inter-quartile range for esmolol infusion rates was approximately 10–60 μg/kg/min, similar to the range of infusion rates observed in our cohort.

We draw attention to the amount of time (approximately 5 h) required at the bedside by study investigators for titration of esmolol, suggesting the importance of placebo controls in future studies, as differential attention from an experienced physician could represent a powerful confounding variable for clinical outcomes in prior trials.

It is possible that our stop event definitions were unduly conservative, and that esmolol intolerance in patients with septic shock is less common than suggested in our pilot study. We cannot address this question on the basis of current evidence, as our patients had positive clinical outcomes even in the presence of apparent intolerance to higher infusion rates of esmolol.

This feasibility study has several limitations, primarily its small size and lack of a placebo control. This study is not intended to support inferences about efficacy. These limitations do not, in our view, constrain the potential relevance of the observations we made. Crucially, we did not feel that enrolling more patients in the present pilot would change our conclusions about the operational intensity of the protocol (affecting feasibility and generalizability for a multi-center trial) and the likely improved tolerance of lower maximal esmolol infusion rates. Crucially, despite its small sample size, we feel that our results strongly suggest equipoise for further trials in a US clinical environment. Such trials should, in our view, evaluate lower maximal infusion rates of esmolol, which would likely improve both the operational intensity of titration protocols and decrease rates of esmolol intolerance.

In terms of current clinical alternatives in the treatment of septic shock, other therapeutic options to decrease catecholamine toxicity are not yet evidence-based. Although ivabradine, a funny channel inhibitor that slows heart rate without affecting contractility, has attracted recent attention, it is not ready for clinical application in septic patients. [56] Nor is there yet sufficient evidence to recommend, e.g., substitution of phenylephrine for norepinephrine to limit exogenous β-adrenergic agonism. [57] Similarly, non-catecholamine vasopressors have not demonstrated efficacy to date for important clinical endpoints. [58–61] Further research is thus indicated to improve approaches to addressing catecholamine toxicity in septic shock.

## Conclusions

Esmolol infusion appears safe with appropriate monitoring in patients with septic shock and persistent tachycardia in a US critical-care environment. Higher infusion rates may be less well tolerated than lower infusion rates, and the intensity of attention during esmolol titration suggests the importance of placebo control in future randomized trials of various dosing strategies, for which strong equipoise exists.

## Additional file

Additional file 1: Online data supplement. (DOCX 5068 kb)

## Abbreviations

APACHE II: Acute Physiology, Age, and Chronic Health Evaluation score, version 2
GLS: Global longitudinal strain
ICU: Intensive care unit
IQR: Inter-quartile range
LV: Left ventricle
LVEF: Left ventricular ejection fraction
MODS: Multiple organ dysfunction syndrome
OFFD: Organ-failure-free days
SOFA: Sequential organ failure assessment
US: United States
