# Supplementary material for: Esmolol infusion in patients with septic shock and tachycardia: a prospective, single-arm, feasibility study
Source: Pilot Feasibility Stud. 2018 Aug 3;4:132. doi: 10.1186/s40814-018-0321-5 (PMC6091011; doi:10.1186/s40814-018-0321-5)
Supplement: Supplementary file 1 — Online data supplement. (DOCX 5068 kb) [file 40814_2018_321_MOESM1_ESM.docx]

ONLINE DATA SUPPLEMENT

Esmolol infusion in patients with septic shock and tachycardia: a prospective, single-arm feasibility study

Samuel Brown, MD MS,^1,2^ Sarah J. Beesley, MD,^1,2^ Colin K. Grissom, MD,^1,2^ Michael J. Lanspa, MD, MS,^1,2^ Emily L. Wilson, MStat,^1^ Samir Parikh, MD,^3^ Todd Sarge, MD,^4^ Daniel Talmor, MD,^4^ Valerie Banner-Goodspeed, MPH,^4^ B. Taylor Thompson, MD,^5^ Sajid Shahul, MD^6^ for the Esmolol to Control Adrenergic Storm in Septic Shock-ROLLIN (ECASSS-R) trial

^1^Pulmonary and Critical Care Medicine, Intermountain Medical Center, Murray, UT

^2^Pulmonary and Critical Care Medicine, University of Utah, Salt Lake City, UT

^3^Nephrology and Vascular Biology, Beth Israel Deaconess Medical Center, Boston, MA

^4^Anesthesia and Critical Care Medicine, Beth Israel Deaconess Medical Center, Boston, MA

^5^Pulmonary and Critical Care Medicine, Massachusetts General Hospital, Boston, MA

^6^Department of Anesthesia, University of Chicago, Chicago, IL

APPENDIX 1. SUPPLEMENTAL METHODS 3

eTable 1. Inclusion and exclusion criteria 3

Approach to identification of patients for comparison 5

Detailed echocardiographic methods 6

Exploration of predictors of possible esmolol intolerance 7

Detailed heart rate variability methods 8

Laboratory methods 8

eTable 2. Pre-enrollment assessment of adequacy of volume expansion 9

eFigure 1. Safety Check 9

eTable 3. Definition of protocol compliance outcomes 9

APPENDIX 2. ESMOLOL TITRATION PROTOCOL 11

APPENDIX 3. NARRATIVE OF DEVELOPMENT OF PROTOCOL 13

APPENDIX 4. SUPPLEMENTAL RESULTS 13

eTable 4. Results of safety check 13

eTable 5. Changes in hemodynamics in association with initial esmolol infusion 14

eTable 6. Echocardiographic parameters 14

eTable 7. Heart rate variability parameters 14

eTable 8. Comparison of patients who did or did not meet STOP event criteria 15

eFigure 2. Dynamic of heart rate and norepinephrine dosing during esmolol infusion 16

eFigure 3. Dynamics of heart rate and cardiac index during esmolol infusion 17

eFigure 4. Changes in left ventricular global longitudinal strain between day 0 and day 1 18

References 19

# APPENDIX 1. SUPPLEMENTAL METHODS

## eTable 1. Inclusion and exclusion criteria

| **Inclusion Criterion** | **Justification/Description** |
| --- | --- |
| Age ≥ 18 years | Children are physiologically distinct from adults |
| Septic shock | Meets consensus criteria:   1. ≥2 SIRS criteria 2. Suspected or confirmed infection 3. Receiving vasopressor infusion to treat hypotension after at least 20ml/kg intravenous crystalloid volume expansion |
| Adequately volume expanded | Designed to include only non-compensatory tachycardia, that which persists after adequate volume expansion. Being no longer fluid responsive requires any one of   1. CVP > 15 mmHg 2. Negative Passive Leg Raise (PLR) maneuver (< 10% increase in cardiac output during PLR) 3. Stroke volume increase < 10% in response to rapid (< 5 min) infusion of at least 250 ml crystalloid solution 4. In patients being passively mechanically ventilated, stroke volume variability (measured by a valid method, including NICOM) < 10% |
| Receiving vasopressor infusion via central venous catheter | At least 60 min of any infusion rate of norepinephrine, epinephrine, vasopressin, or phenylephrine |
| Current or imminent placement of arterial catheter | Allows continuous measurement of blood pressures during esmolol infusion and is standard in the study ICU. |
| Tachycardia (>90/min) | Heart rate must remain > 90/min for at least one hour, *while* the patient is receiving vasopressors |
|  |  |

| **Exclusion Criterion** | **Justification/Description** |
| --- | --- |
| Cardiogenic shock | Safety consideration**: β**-blockade may worsen preexisting cardiogenic shock. Cardiogenic shock defined as *any one* of   1. Cardiac Index ≤ 2 L/min/m^2^ 2. ScvO_2_ ≤ 60% 3. Ejection Fraction ≤ 25% 4. Current infusion of any dose of dobutamine, milrinone, or dopamine 5. Current infusion of epinephrine to treat clinically diagnosed cardiogenic shock. |
| Preexisting heart block | Safety consideration**: β**-blockade may worsen heart block.  Defined as any of the following   1. Sick sinus syndrome 2. PR interval > 200 msec 3. Any current evidence or history of Grade 2 or Grade 3 heart block 4. Pacemaker or plans to place a pacemaker |
| Atrial fibrillation at time of enrollment | Substantial noise associated with exposure, hemodynamic measurements, and outcomes; may require independent heart rate control or cardioversion. |
| Current nodal blockers or anti-arrhythmic agents | Safety consideration: risk of excess nodal blockade. Increased experimental noise.  Receipt of any of the following within 3 half lives (see full list in Appendix)   1. Non-dihydropyridine calcium channel blockers 2. Digitalis 3. Anti-arrhythmics (e.g., amiodarone, dronedarone, mexiletine) 4. Beta blockers |
| Receiving clonidine, guanfacine, or moxonidine | Safety issue |
| Moderate or severe pulmonary hypertension | Possible safety consideration; risk of hemodynamic deterioration.  Defined as a documented history of prior right heart catheterization or current evidence on TTE of **any** of the following.   1. mPAP ≥ 35 mmHg 2. SPAP ≥ 60 mmHg |
| Allergy to esmolol or vehicle | Rare, but required for patient safety. |
| Admitted for hospice or comfort care | Incomplete and biased data on both exposure and outcome. |
| Inability to obtain consent within 48 hours of ICU admission | This study does not qualify for waiver of consent |
| Active pregnancy or nursing | Physiology of pregnant patients distinct; potential risks to fetus unknown; drug levels in breast milk. |
| Prisoners | Septic shock is not specific to prisoners, so ethical concerns emphasize the protection of autonomy. |
| Previously enrolled in this trial | Violation of the independence assumption. |
| Pheochromocytoma or status asthmaticus | Safety issue associated with beta blockade |
| Hemoglobin < 7 gm/dl | Exclude anemia as cause of tachycardia; avoid risk of impaired oxygen delivery related to anemia |
| Cardiovascular collapse | Safety consideration; failure to achieve 65mmHg MAP with vasopressors. |
| Cardiac arrest within 24 hours | Safety consideration |
| Worse than moderate aortic stenosis | Known aortic stenosis, with any of (1) mean gradient ≥ 40 mmHg OR (2) maximum gradient ≥ 60mmHg OR (3) aortic valve area ≤ 1.0cm^2^ OR (4) aortic valve area index ≤ 0.85cm^2^/m^2^ body surface area. This is a hypothetical safety consideration. |
| Worse than mild mitral stenosis | Known mitral stenosis, with any of (1) valve area ≤ 1.5 cm^2^ OR mean gradient ≥ 5 mmHg. This is a hypothetical safety consideration. |

## Approach to identification of patients for comparison

Potential comparison patients were drawn from two overlapping prospectively identified cohorts of patients with sepsis or septic shock in whom echocardiograms were available in the first day of ICU admission.(1, 2) These patients were prospectively identified as part of an ongoing cohort of patients identified using then-current sepsis consensus guidelines as were applied for ECASSS-R. We used patients from research cohorts rather than a clinical cohort to improve the face validity of comparison, as clinical trial populations commonly differ from clinical population cohorts.(3) Among these cohorts, comparison was restricted to patients meeting the specific eligibility criteria of ECASSS-R, as determined by chart review.

We attempted a direct match, 1:2, based on an exact match for source of sepsis (dichotomized to urinary vs. non-urinary sepsis) and use of mechanical ventilation, while for other matching variables (i.e., age, admission APACHE II score, heart rate and norepinephrine equivalent dose [other vasopressors converted according to standard equivalencies(4)] at time of esmolol initiation), we identified the closest match (Euclidean distance after standardization), with certain constraints to avoid imbalance on a specific variable. The constraints were admission APACHE II score within 5 points, heart rate within 15 beats/min, minimum norepinephrine equivalent >0.08 mcg/kg/min. Because esmolol infusion was initiated at different times after initiation of vasopressor infusion, among match patients we identified the time after initiation of vasopressor onset that corresponded with the time-lag for the corresponding esmolol patient. We used this approach to avoid comparison of heart rate and norepinephrine infusion rate at arbitrary time points. Unfortunately, given data sparsity we were unable to achieve a 1:2 match for all patients. We therefore report, without p values, the overall comparison cohort of 105 patients who met ECASSS-R eligibility criteria.

## Detailed echocardiographic methods

Transthoracic echocardiograms (TTEs) were obtained using Philips IE33 (Bothell, WA) ultrasound machines. All TTEs were read by a Level-II cardiac sonographer who is a Fellow of the American Society of Echocardiography. Our primary variable of interest was left ventricular (LV) global longitudinal strain (GLS). Strain represents the amount of deformation within the heart, conventionally measured on the basis of endocardial movement. While historically strain was measured with tissue Doppler, in recent years it has been measured with speckle tracking echocardiography. Strain has been validated in multiple settings, including patients with coronary disease and septic patients.(5-11) Strain appears superior to LV ejection fraction (EF) as a measure of cardiac dysfunction.(12, 13)

Left ventricular GLS was measured from the 2-, 3-, and 4-chamber views in the apical window using Tomtec™ automated strain software, which has been well validated.(14-17) We report endocardial GLS. We defined abnormal GLS as >-19%,(18) despite small studies arguing for a threshold closer to -17%.(11, 19) (By convention, GLS is measured as a negative number, so worse strain is numerically greater than better strain even though the absolute value of worse strain is smaller than the absolute value of better strain.) We rejected images due to poor image quality if we could not speckle-track two or more adjacent segments in the apical four-chamber view.

## Exploration of predictors of possible esmolol intolerance

Acknowledging that small numbers limit reliable inference, we nevertheless sought to understand whether patients who developed possible esmolol intolerance differed in some way from those who did not. We considered age, APACHE II score, norepinephrine dose at esmolol initiation, arterial elastance, LVEF, ScvO_2_, LV GLS, stroke volume from initial echocardiogram, total volume of crystalloid infused before enrollment, and mean arterial pressure. We tentatively used Wilcoxon rank sum to compare distributions of these variables between patients who tolerated esmolol infusion and those who did not, considering p ≤ 0.1 to suggest a relationship of potential interest.

## Detailed heart rate variability methods

The signal for the lead II EKG from Philips Intellivue™ bedside monitor was obtained via the slave (cardioversion) port and digitized using a digital-analog converter (National Instruments, Austin, TX) with a sampling frequency of 500 Hz. The digitized sample was then processed in the Continuous Individual Multiorgan Variability Analysis (CIMVA™; Dynamic Analysis Laboratory, Ottawa) software(20) using the Elgendi peak identification algorithm.(21) Attributes of the heart rate variability were calculated according to CIMVA standard methods. We measured sequential 30-minute windows, without overlap. We generated a full profile of complexity measures (including time-, frequency-, and complexity-domain metrics). In this study, we evaluated the normalized LF:HF ratio and the ratio of fractal exponents from detrended fluctuation analysis, metrics we have validated in prior work in sepsis.(22, 23)

## Laboratory methods

Plasma samples were assayed for Angiopoietin-2 levels at enrollment and day 1. Samples were placed in citrated tubes, spun to remove supernatant and aliquoted into cryovials. The Angiopoietin-2 concentration in plasma (1:25 dilution) was measured with a commercial human ELISA (DANG20, R&D Systems, Minneapolis, MN) in accordance with the instructions of the manufacturer. The R^2^ of the standard curve was 0.99. The coefficient of variation in the analyzing laboratory is < 10%.

## eTable 2. Pre-enrollment assessment of adequacy of volume expansion

| **Modality** | **Threshold** |
| --- | --- |
| Central venous catheter | Central venous pressure > 15 mmHg |
| Passive Leg Raise maneuver | <10% increase in cardiac index |
| Graded volume expansion challenge | <10% increase in cardiac index after rapid infusion of at least 250ml intravenous crystalloid |
| Clinical assessment | Treating physician deems volume expansion adequate |
| Dynamic measures among passively ventilated patients (at least 8ml/kg tidal volume, normal sinus rhythm) | <10% stroke volume variability (or equivalent, depending on modality) |

## eFigure 1. Safety Check


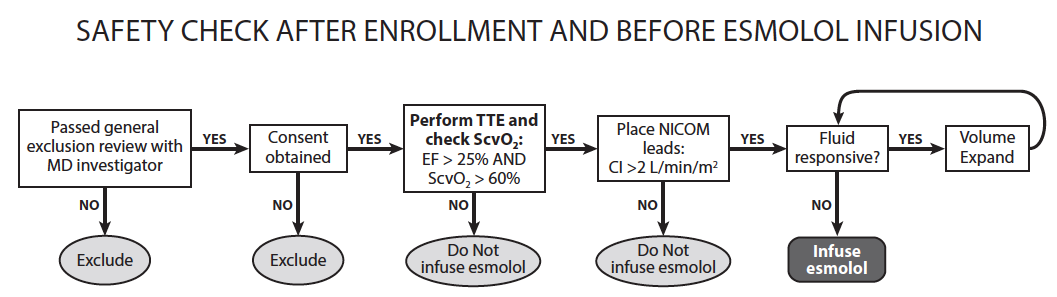


| eTable 3. Definition of protocol compliance outcomes | |
| --- | --- |
| **Parameter** | **Compliance definition** |
| *Safety check* | 100% compliance with all aspects. |
| *Esmolol titration protocol* | Each hour (the closest value of heart rate to the hour) during the esmolol infusion will be determined to be “in range” or “out of range,” with 3bpm margin for compliance (i.e., heart rate 77 to 93bpm). The initiation and cessation of esmolol will also be included as a timepoint for evaluation of compliance. Protocol compliance is considered adequate where overall compliance on hourly checks is >80%. |
| *STOP events* | 100% compliance, metrics defined by  --30min moving average of cardiac index below threshold  --30min moving average of norepinephrine infusion rate above threshold  --Clinician determines that shock has substantially worsened on esmolol  --Grade II or Grade III heart block |

# APPENDIX 2. ESMOLOL TITRATION PROTOCOL

**Fundamental rules for esmolol titration**:

The target heart rate is 85 bpm. Start esmolol infusion at 20 mcg/kg/min, without bolus, if HR ≥ 100 bpm. If HR >90 bpm and <100 bpm, start esmolol infusion at 10 mcg/kg/min, without bolus. Increase by 20 mcg/kg/min every 20 minutes as long as HR > 90 bpm, to a maximum dose of 100 mcg/kg/min. If HR < 80 bpm and > 70 bpm, decrease infusion rate by 10 mcg/kg/min; if HR ≤ 70 bpm and > 60 bpm, decrease infusion rate by 20 mcg/kg/min. Stop esmolol infusion whenever a STOP EVENT occurs. Esmolol infusion is completed 3 hours after vasopressor infusions have stopped. The up-titrations can be smaller than stipulated if the clinician and/or investigator feels that a small up-titration is indicated. Up-titrations should not occur during a REASSESS VOLUME STATUS event.

Esmolol should be preferentially infused centrally; where no port is available, esmolol should **not be** infused into a small peripheral vein or via butterfly catheter.

STOP EVENTS:

1. If HR is ever ≤ 60 bpm, immediately stop infusion for 20 minutes, then restart at half the previous infusion rate, rounded to nearest 10 mcg/kg/min. Do not resume infusion until HR > 70 bpm.
2. If vasopressor dose increases by 0.2 mcg/kg/min norepinephrine (or equivalent) AND doubles within 60 min despite adequate volume expansion, STOP the esmolol infusion. Alternatively, if the treating clinician believes that the shock is worsening significantly, STOP the esmolol infusion. Do not resume without explicit approval of study physician.
3. If cardiogenic shock develops (ScvO_2_ ≤ 60% OR LV ejection fraction ≤ 25% OR Cardiac Index ≤ 2.0 L/min/m^2^), STOP the esmolol infusion. Do not resume without explicit approval of study physician.
4. If clinically apparent bronchospasm develops, STOP the esmolol infusion. Do not resume.

REASSESS VOLUME STATUS EVENTS:

When markers of potential preload deficit occur (urine output < 0.5 ml/kg/hr OR vasopressor dosage increase), immediately EVALUATE FOR VOLUME RESPONSIVENESS. A vasopressor dosage increase is defined as at least 0.05 mcg/kg/min absolute AND 20% relative increase in the norepinephrine (or equivalent) infusion.

Titrate vasopressors as per local practice to maintain MAP ≥ 65mmHg. Norepinephrine is the preferred primary vasopressor. Consider adding vasopressin when norepinephrine infusion rate is > 0.2 mcg/kg/min, and consider adding epinephrine and stress-dose steroids at > 0.5 mcg/kg/min of norepinephrine. Phenylephrine use is discouraged.


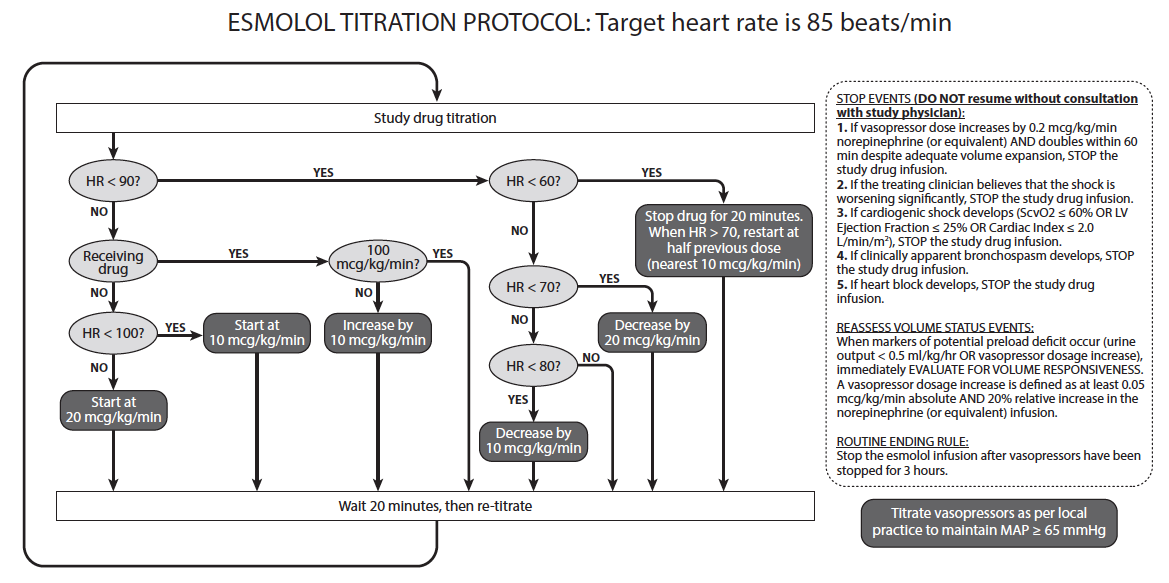


# APPENDIX 3. NARRATIVE OF DEVELOPMENT OF PROTOCOL

On the basis of experience acquired during the study, we modified the study protocol to (a) stop esmolol infusion during trips to the operating room, (b) require another review of the adequacy of volume expansion and cardiac function after a return from the operating room, (c) adjust the default initial infusion rate for patients with heart rate 90-100/min, (d) further refine eligibility criteria (ultimately resulting in the criteria displayed in eTable 1; notable additions included significant valvular disease and recent cardiac arrest). All enrolled patients met the final eligibility criteria.

After seven patients had been enrolled, given feedback from clinical and research staff regarding the intensity of the titration protocol as well as the suggestion of intolerance at higher rates of esmolol infusion, we elected to transition to a substantially simplified, lower-dose infusion protocol for a second rollin pilot.

# APPENDIX 4. SUPPLEMENTAL RESULTS

## eTable 4. Results of safety check

| **Variable** | **Mean (standard deviation)** |
| --- | --- |
| ScvO_2_ (%) | 76 (5) |
| LVEF (%) | 55 (8) |
| Cardiac index (L/min/m2) | 3.6 (1.3) |
| Adequacy of volume expansion (%)* | 100% |
| Passive leg raise (n=4)  Graded volume expansion (n=4)  Central venous catheter (n=1)  Clinical assessment (n=1)  Dynamic measures on ventilator (n=0) | Change in cardiac index: 3.2 (4.0) %  Change in cardiac index: 5.3 (1.5) %  CVP: 18mmHg  Stroke volume variability: NA |
| *Two patient were assessed with multiple modalities, so modalities sum to 10 rather than 7. | |

## eTable 5. Changes in hemodynamics in association with initial esmolol infusion

| **Variable** | **Change** |
| --- | --- |
| Cardiac index (L/min/m^2^ BSA) | -0.1 L/min/m^2^ BSA |
| Heart Rate (min^-1^) | -6 min^-1^ |
| Norepinephrine infusion rate (mcg/kg/min) | +0.02 mcg/kg/min |
| Mean arterial pressure (mmHg) | -4 mmHg |
| *Average effect observed with an esmolol titration of 10 mcg/kg/min, at initial doses  BSA: body surface area | |

## eTable 6. Echocardiographic parameters

| **Parameter** | **Enrollment TTE** | **Day 1 TTE** | **P value** |
| --- | --- | --- | --- |
| LV GLS (%) | -11.5 (-19.6, -9.4) | -15.8 (-20.1, -13.5) | 0.62 |
| LV EF (%) | 59 (48, 60) | 56 (49, 62) | 0.8 |
| LV tMPI | 0.58 (0.50, 0.62) | 0.56 (0.46, 0.84) | 1 |
| LV: left ventricular; GLS: global longitudinal strain; EF: ejection fraction; tMPI: tissue Myocardial Performance Index | | | |

## eTable 7. Heart rate variability parameters

| **Parameter** | **Enrollment** | **Followup** | **P value** |
| --- | --- | --- | --- |
| LF:HF ratio | 0.78 (0.38, 3.4) | 0.54 (0.45, 0.70) | 0.62 |
| Detrended fluctuation analysis |  |  |  |
| α1 | 0.60 (0.44, 0.89) | 0.79 (0.70, 0.87) | 0.80 |
| α2 | 0.96 (0.71, 1.03) | 0.70 (0.56, 0.90) | 0.21 |
| α1/ α2 | 0.84 (0.53, 0.94) | 1.08 (0.82, 1.12) | 0.38 |
| Shannon Entropy | 4.4 (4.0, 4.6) | 3.8 (3.5, 4.8) | 0.46 |
| LF:HF ratio: ratio of low frequency to high frequency power from spectral density, calculated by the Lomb-Scargle method | | | |

## eTable 8. Comparison of patients who did or did not meet STOP event criteria

| Variable | No STOP event (N=4) | STOP event (N=3) | p-value* |
| --- | --- | --- | --- |
| Age (years) | 51 (42-61) | 30 (26-46) | 0.38 |
| Maximum esmolol infusion rate (mcg/kg/min) | 25 (18-35) | 50 (50-75) | **0.10** |
| Admission APACHE II | 23 (23-25) | 28 (27-36) | 0.21 |
| Norepinephrine dose at esmolol initiation^a^ (mcg/kg/min) | 0.14 (0.10-0.20) | 0.22 (0.21-0.30) | 0.21 |
| Arterial elastance^b^ (mmHg/ml) | 1.0 (0.9-1.1) | 1.8 (1.6-2.2) | **0.05** |
| Left ventricular EF^b^ (%) | 59 (57-60) | 46 (40-54) | 0.37 |
| ScvO_2_^b^ (%) | 78 (77-80) | 73 (70-75) | 0.11 |
| Left ventricular GLS^b^ | -19.6 (-22.1- -15.6) | -9.7 (-10.6- -8.1) | 0.22 |
| Shannon entropy of heart rate^b^ | 4.4 (4.3-4.7) | 4.1 (4.0-4.4) | 0.60 |
| DFA ratio^b^ | 0.53 (0.43-0.78) | 0.90 (0.87-0.94) | 0.38 |
| Angiopoietin-2^b^ (ng/ml) | 23 (18-38) | 17 (14-26) | 0.86 |
| Stroke volume^b^ | 75 (70-75) | 39 (32-46) | **0.05** |
| Fluid administered prior to enrollment (L) | 6.2 (3.2-9.0) | 3.5 (3.5-6.4) | 0.59 |
| MAP^a^ (mmHg) | 70 (68-75) | 69 (67-70) | 0.59 |
| ^a^At time of esmolol initiation  ^b^At time of enrollment  EF: ejection fraction; GLS: global longitudinal strain; DFA ratio: ratio of fractal exponents from detrended fluctuation analysis; MAP: mean arterial pressure. | | | |

## eFigure 2. Dynamic of heart rate and norepinephrine dosing during esmolol infusion


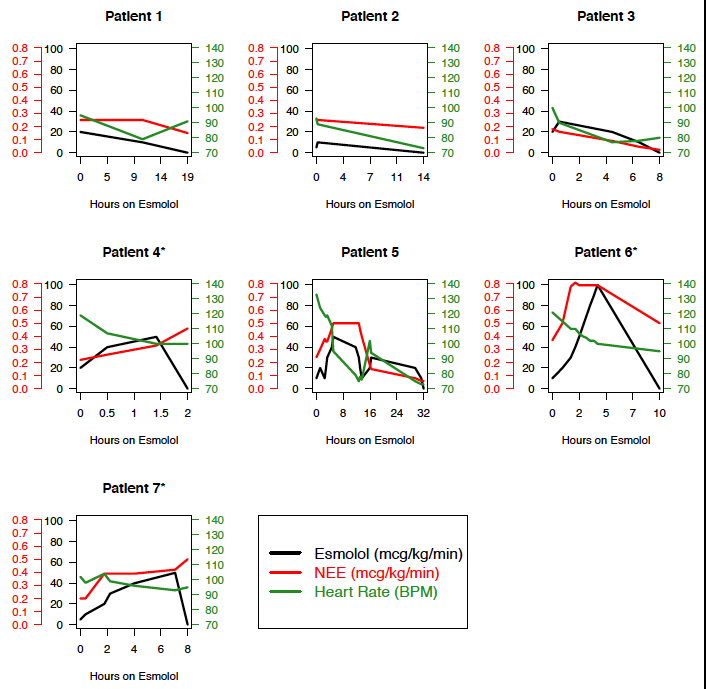


*

## eFigure 3. Dynamics of heart rate and cardiac index during esmolol infusion


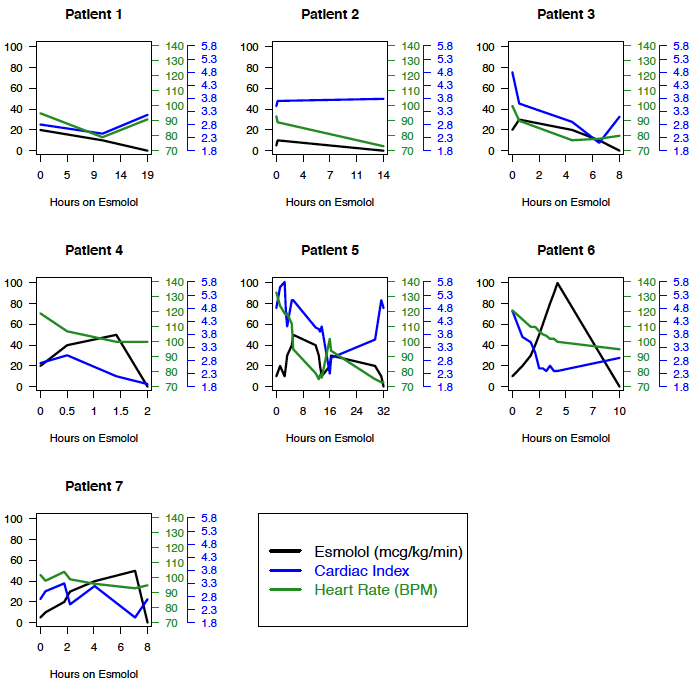


## eFigure 4. Changes in left ventricular global longitudinal strain between day 0 and day 1


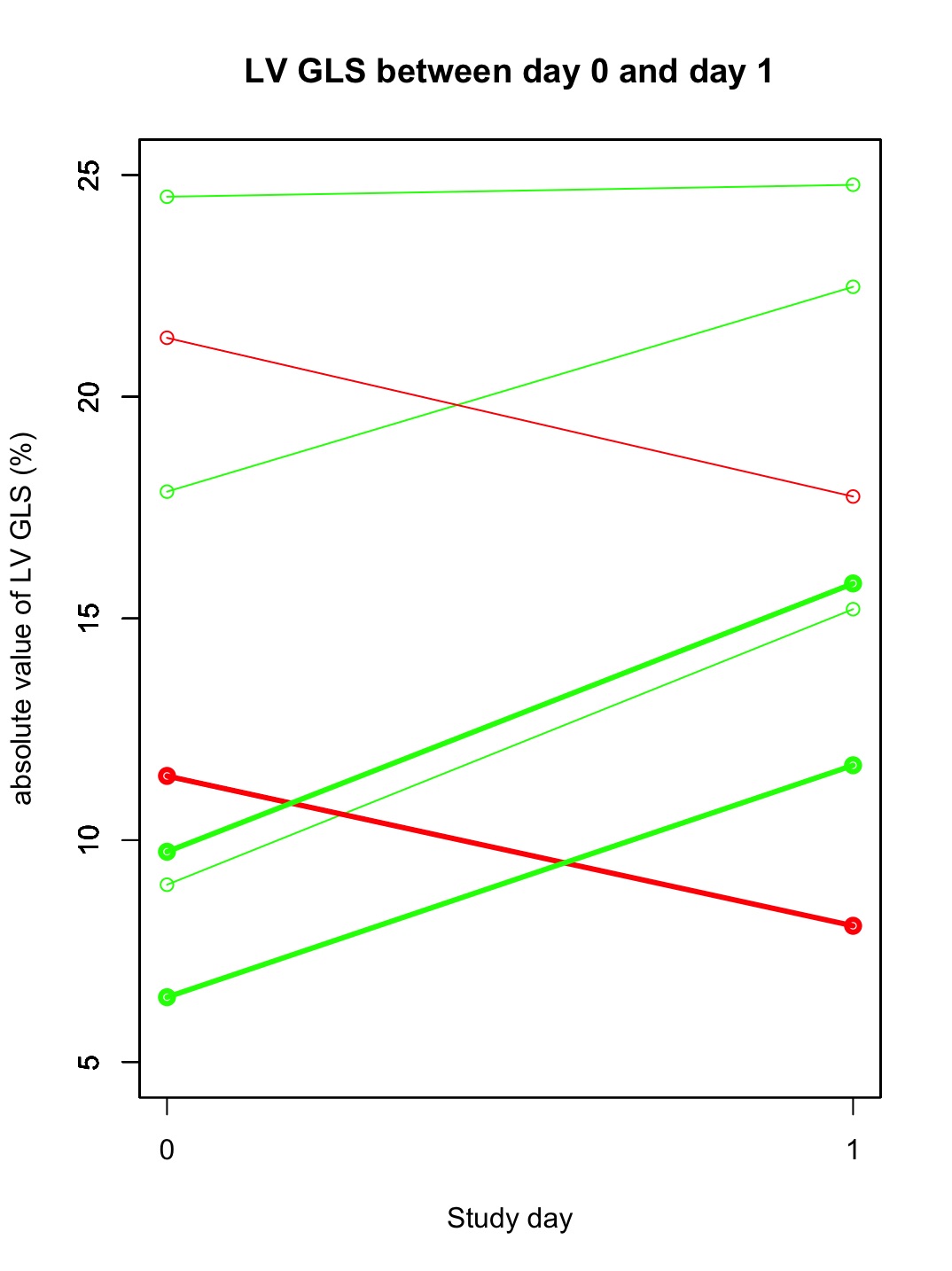
Green indicates improvement in strain, red indicates worsening in strain. Bold lines indicate patients who had a STOP event.

# References

1. Lanspa MJ, Gutsche AR, Wilson EL, Olsen TD, Hirshberg EL, Knox DB, Brown SM, Grissom CK. Application of a simplified definition of diastolic function in severe sepsis and septic shock. *Crit Care* 2016; 20: 243.

2. Lanspa MJ, Shahul S, Hersh A, Wilson EL, Olsen TD, Hirshberg EL, Grissom CK, Brown SM. Associations among left ventricular systolic function, tachycardia, and cardiac preload in septic patients. *Annals of intensive care* 2017; 7: 17.

3. Kennedy-Martin T, Curtis S, Faries D, Robinson S, Johnston J. A literature review on the representativeness of randomized controlled trial samples and implications for the external validity of trial results. *Trials* 2015; 16: 495.

4. Brown SM, Lanspa MJ, Jones JP, Kuttler KG, Li Y, Carlson R, Miller RR, Hirshberg EL, Grissom CK, Morris AH. Survival after shock requiring high-dose vasopressor therapy. *Chest* 2013; 143: 664-671.

5. Geyer H, Caracciolo G, Abe H, Wilansky S, Carerj S, Gentile F, Nesser HJ, Khandheria B, Narula J, Sengupta PP. Assessment of myocardial mechanics using speckle tracking echocardiography: fundamentals and clinical applications. *J Am Soc Echocardiogr* 2010; 23: 351-369; quiz 453-355.

6. Mondillo S, Galderisi M, Mele D, Cameli M, Lomoriello VS, Zaca V, Ballo P, D'Andrea A, Muraru D, Losi M, Agricola E, D'Errico A, Buralli S, Sciomer S, Nistri S, Badano L. Speckle-tracking echocardiography: a new technique for assessing myocardial function. *Journal of ultrasound in medicine : official journal of the American Institute of Ultrasound in Medicine* 2011; 30: 71-83.

7. Nesbitt GC, Mankad S, Oh JK. Strain imaging in echocardiography: methods and clinical applications. *The international journal of cardiovascular imaging* 2009; 25 Suppl 1: 9-22.

8. Yagmur J, Sener S, Acikgoz N, Cansel M, Ermis N, Karincaoglu Y, Tasolar H, Karakus Y, Pekdemir H, Ozdemir R. Subclinical left ventricular dysfunction in Behcet's disease assessed by two-dimensional speckle tracking echocardiography. *Eur J Echocardiogr* 2011; 12: 536-541.

9. Shahul S, Rhee J, Hacker MR, Gulati G, Mitchell JD, Hess P, Mahmood F, Arany Z, Rana S, Talmor D. Subclinical left ventricular dysfunction in preeclamptic women with preserved left ventricular ejection fraction: a 2D speckle-tracking imaging study. *Circ Cardiovasc Imaging* 2012; 5: 734-739.

10. Shahul S, Gulati G, Hacker MR, Mahmood F, Canelli R, Nizamuddin J, Mahmood B, Mueller A, Simon BA, Novack V, Talmor D. Detection of Myocardial Dysfunction in Septic Shock: A Speckle-Tracking Echocardiography Study. *Anesth Analg* 2015; 121: 1547-1554.

11. Lanspa MJ, Pittman JE, Hirshberg EL, Wilson EL, Olsen T, Brown SM, Grissom CK. Association of left ventricular longitudinal strain with central venous oxygen saturation and serum lactate in patients with early severe sepsis and septic shock. *Crit Care* 2015; 19: 304.

12. Hoit BD. Strain and strain rate echocardiography and coronary artery disease. *Circ Cardiovasc Imaging* 2011; 4: 179-190.

13. Voigt JU, Exner B, Schmiedehausen K, Huchzermeyer C, Reulbach U, Nixdorff U, Platsch G, Kuwert T, Daniel WG, Flachskampf FA. Strain-rate imaging during dobutamine stress echocardiography provides objective evidence of inducible ischemia. *Circulation* 2003; 107: 2120-2126.

14. Bagger T, Sloth E, Jakobsen CJ. Left ventricular longitudinal function assessed by speckle tracking ultrasound from a single apical imaging plane. *Critical care research and practice* 2012; 2012: 361824.

15. Williams LK, Urbano-Moral JA, Rowin EJ, Jamorski M, Bruchal-Garbicz B, Carasso S, Pandian NG, Maron MS, Rakowski H. Velocity vector imaging in the measurement of left ventricular myocardial mechanics on cardiac magnetic resonance imaging: correlations with echocardiographically derived strain values. *J Am Soc Echocardiogr* 2013; 26: 1153-1162.

16. Amundsen BH, Helle-Valle T, Edvardsen T, Torp H, Crosby J, Lyseggen E, Stoylen A, Ihlen H, Lima JA, Smiseth OA, Slordahl SA. Noninvasive myocardial strain measurement by speckle tracking echocardiography: validation against sonomicrometry and tagged magnetic resonance imaging. *J Am Coll Cardiol* 2006; 47: 789-793.

17. Knackstedt C, Bekkers SC, Schummers G, Schreckenberg M, Muraru D, Badano LP, Franke A, Bavishi C, Omar AM, Sengupta PP. Fully Automated Versus Standard Tracking of Left Ventricular Ejection Fraction and Longitudinal Strain: The FAST-EFs Multicenter Study. *J Am Coll Cardiol* 2015; 66: 1456-1466.

18. Yingchoncharoen T, Agarwal S, Popovic ZB, Marwick TH. Normal ranges of left ventricular strain: a meta-analysis. *J Am Soc Echocardiogr* 2013; 26: 185-191.

19. Orde SR, Pulido JN, Masaki M, Gillespie S, Spoon JN, Kane GC, Oh JK. Outcome prediction in sepsis: speckle tracking echocardiography based assessment of myocardial function. *Crit Care* 2014; 18: R149.

20. Seely AJ, Green GC, Bravi A. Continuous Multiorgan Variability monitoring in critically ill patients--complexity science at the bedside. *Conf Proc IEEE Eng Med Biol Soc* 2011; 2011: 5503-5506.

21. Elgendi M. Fast QRS detection with an optimized knowledge-based method: evaluation on 11 standard ECG databases. *PLoS One* 2013; 8: e73557.

22. Brown SM, Tate Q, Jones JP, Knox DB, Kuttler KG, Lanspa M, Rondina MT, Grissom CK, Behera S, Mathews VJ, Morris A. Initial fractal exponent of heart rate variability is associated with success of early resuscitation in patients with severe sepsis or septic shock: a prospective cohort study. *J Crit Care* 2013; 28: 959-963.

23. Brown SM, Sorensen J, Lanspa M, Rondina MT, Grissom CK, Shahul S, Mathews VJ. Multi-complexity measures of heart rate variability and the effect of vasopressor titration: a prospective cohort study of patients with septic shock. *BMC Infectious Diseases* 2016; In press.
